# Medication-related osteonecrosis of the jaw: knowledge and perceptions of medical professionals on the usage of bone modifying agents and dental referrals

**DOI:** 10.25122/jml-2021-0085

**Published:** 2022-03

**Authors:** Shruthi Acharya, Vathsala Patil, Vineetha Ravindranath, Adarsh Kudva, Krithi Nikhil

**Affiliations:** 1. Department of Oral Medicine and Radiology, Manipal College of Dental Sciences, Manipal, Manipal Academy of Higher Education, Manipal, Karnataka, India; 2. Department of Oral and Maxillofacial Surgery, Manipal College of Dental Sciences, Manipal, Manipal Academy of Higher Education, Manipal, Karnataka, India; 3. Department of Public Health Dentistry, SDM College of Dental Sciences and Hospital, Shri Dharmasthala Manjunatheshwara University, Dharwad, Karnataka, India

**Keywords:** angiogenic inhibitor, antiresorptive agents, awareness, bisphosphonates, osteonecrosis, BMA – Bone modifying agents, MRONJ – Medication-related osteonecrosis of the jaw, SRE –Skeletal Related Events, IV – Intravenous therapy, BRONJ – Bisphosphonate-related osteonecrosis of the jaw

## Abstract

Bone modifying agents (BMA) like bisphosphonates, antiangiogenic, and antiresorptive agents are widely used to manage bone diseases and cancer. Medication-related osteonecrosis of the jaw (MRONJ) is a potentially serious complication seen in patients on BMA therapy. Dental infection is one of the primary risk factors for MRONJ manifestation; hence its complete removal before initiation of BMA is significant. This can be achieved when a medical professional understands MRONJ and its risk factors and implements timely and regular dental referrals. This multicentre study was performed to assess the knowledge about MRONJ and awareness about the implementation of dental referrals among medical professionals. A custom-designed questionnaire tool was designed and validated by a pilot study. 450 practitioners from 6 medical schools and private practitioners in and around the district were surveyed. The results were analyzed using descriptive statistics. 63.5% (n=80) of the respondents prescribed bisphosphonates at a frequency of 0–5 times in a month. However, 62% (n=78) of the practitioners could correctly indicate the most appropriate definition of MRONJ. Only 49.2% (n=62) of them considered dental consultation mandatory. 73% (n=92) of the practitioners were unaware of management guidelines. There exists a significant gap in the knowledge-based applications in the management of MRONJ. Lack of referrals to dentists before BMA therapy can be a pivotal factor in patient morbidity. Practitioners prescribing BMA should advise patients about regular dental visits and monitor for symptoms of MRONJ.

## INTRODUCTION

Medication-related osteonecrosis of the jaw (MRONJ) is a potentially serious complication seen in patients on bone-modifying agents (BMA), principally bisphosphonates, antiangiogenic, and antiresorptive agents [1]. MRONJ requires complex management, and the associated morbidity and challenges in treatment warrant a preventive care intervention [2]. Along with risk factors like duration of drug intake, concomitant use of corticosteroids, and route of administration, invasive dental procedures like dental extractions are major risk factors for MRONJ [3]. Therefore, complete removal or stabilization of oral infections before initiating these medications is an ideal preventive care strategy [3, 4].

A study on general medical practitioners and pharmacists in North East England found that professional groups had limited knowledge and awareness about MRONJ. Due to the complex medical histories of patients, practitioners often overlook advice related to the risk and prevention of this condition [2]. Masson *et al.* identified that only 11.8% of general medical practitioners and 9.7% of pharmacists advised patients to inform their dentist about their bisphosphonate intake [5]. The study performed by Patik V *et al.* amongst dentists to evaluate their knowledge and awareness regarding MRONJ and its management showed that most dentists also lack adequate information regarding MRONJ and its management [6].

In a SWOGO702 trial, the cumulative incidence of MRONJ at 3 years was 2.8% [4]. With the increasing use of bone modifying agents, this is expected to escalate further. Good knowledge of the risk factors and consequences among dentists and medical practitioners is an important determinant for preventing and managing this condition. We identified a gap in referral patterns of patients with BMAs, who were never referred for dental evaluation, and this was not explicitly explored in previous studies. Therefore, this multicentre survey was conducted among the medical practitioners from the hospitals in and around our district to assess their knowledge and perception about MRONJ and the awareness and implementation of dental referrals to patients being advised with BMAs.

## MATERIAL AND METHODS

A multicentric questionnaire was conducted from March 2019 to November 2019 involving medical practitioners of different experience levels and groups who often prescribe bisphosphonates and other BMA and antiangiogenic agents to patients. This included orthopedics, surgeons, general/internal medicine practitioners, and oncologists. Around 450 medical practitioners from 6 medical schools (tertiary healthcare centers) and private practitioners in and around the district were approached and surveyed through a google form questionnaire sent through emails. Some of them were approached in person during their annual meetings and continuing medical education programs. Considering the population of 500 health professionals in five centers, with a 10% margin of error and confidence level of 95%, we arrived at a sample size of 81 responders.

The questionnaire was initially pilot tested in the same study group for feasibility. The interpretations were validated among 20 participants. The initial section of the questionnaire gathered their demographics, specialty, designation, and years of experience. The next section of the questionnaire assessed the main indications and frequency of prescribing bisphosphonates, the most common form of bisphosphonates prescribed, awareness about the oral side effects of bisphosphonates and other bone modifying agents, and whether they have come across osteonecrosis cases as side effects of these medications. Their knowledge regarding the definition of MRONJ and awareness about the concept of "drug holiday for prevention of MRONJ" was also included in the questionnaire. A question was also framed to ascertain their understanding regarding the importance of dental consultation before starting bisphosphonates, BMAs, and antiangiogenic medications. The responses collected were entered into a Microsoft Excel spreadsheet (Microsoft Corp., Redmond, WA) and were analyzed using SPSS software version 20 (IBM, Armonk, NY).

### Statistical Analysis

Descriptive analysis was performed to identify the percentage of responses to each question. Chi-square analysis was done to assess the difference in the proportion of responses among dentists. SPSS version 19 was used. P-value <0.05 was considered statistically significant.

## RESULTS

The questionnaire was distributed to 450 medical practitioners, out of which 126 responses were obtained. 38 respondents were general medicine practitioners, 72 were orthopedic surgeons, 14 were radiation oncologists, 1 general surgery, and 1 did not reveal his field of specialty. Among the respondents, 28 were assistant professors, 13 were associate professors, 51 were senior residents, and 20 were professors with more than 15 years of experience, and 9 were junior residents. Table 1 provides demographic details of the participants who responded to the questionnaire. There was a statistically significant difference among the number of participants based on specialty and designation, with a P-value of <0.001 for Speciality and Designation and 0.45 for Years of experience.

**Table 1.** Demographic characteristics of participants.

	Frequency	Percentage
**Specialty**		
Not responded	1	0.8
General/Internal Medicine	38	30.2
Surgeons	1	0.8
Orthopedics	72	57.1
Oncologists	14	11.1
Total	126	100.0
**Designation**		
Not responded	1	0.8
Assistant Professor	28	22.2
Associate Professor	13	10.3
Post Graduate	51	40.5
Professor	20	15.9
Reader	4	3.2
Senior Resident	9	7.1
Total	126	100.0
**Years of experience**		
Not responded	9	7.1
Less than 5 years	63	50.0
More than 5 years	54	42.9
Total	126	100.0

63.5% (n=80) of the respondents stated that they prescribe antiresorptive agents and/or bisphosphonates about 0–5 times in a month. 20.6% (n=26) said they prescribed around 5–10 times in a month, and only 13.5% (n=17) prescribed more than 10 times in a month. Maximum prescription of bisphosphonates was indicated for osteoporosis, followed by metastatic bone tumor and multiple myeloma. The majority of practitioners prescribed bisphosphonates through the oral route (46.8%, n=59), 19.8% (n=25) prescribed through the IV route, and 30.2% (n=38) of them prescribed bisphosphonates in both forms. Table 2 describes the distribution of correct answers and missing responses to each question.

**Table 2.** Distribution of correct answers and missing responses to each question.

	Frequency	Percent
**How often do you prescribe bisphosphonates in a month?**		
0–5 times	80	63.5
5–10 times	26	20.6
>10 times	17	13.5
not responded	3	2.4
**Which of the following is the side effect of Bisphosphonates in patients undergoing the dental surgical procedure?**		
Correct answer	96	76.2
Wrong answer	26	20.6
Not responded	4	3.2
**Bisphosphonates are commonly prescribed by you for**		
Breast Cancer	3	2.4
Metastatic Bone Tumors	7	5.6
Multiple Myeloma	6	4.8
Osteoporosis	76	60.3
Breast Cancer and Metastatic Bone Tumors	4	3.2
Breast Cancer, Metastatic Bone Tumors, and Multiple Myeloma	2	1.6
Breast Cancer, Metastatic Bone Tumors and Multiple Myeloma, and Osteoporosis	3	2.4
Breast Cancer Metastatic Bone Tumors Osteoporosis	3	2.4
Breast Cancer Multiple Myeloma	2	1.6
Breast Cancer and Osteoporosis	4	3.2
Metastatic Bone Tumors Multiple Myeloma Osteoporosis	6	4.8
Metastatic Bone Tumors, Osteoporosis	6	4.8
Multiple Myeloma, Osteoporosis	1	0.8
Not responded	3	2.4
**Which form of bisphosphonates do you prescribe the most?**		
Oral	59	46.8
Intravenous	25	19.8
Both	38	30.2
Not responded	4	3.2
**What is MRONJ? Please tick the most appropriate answer**		
Correct answer	78	61.9
Wrong answer	42	33.3
Not responded	6	4.8

When the practitioners were asked to select the dental side effects of these medications, 76.2% (n=96) of them selected the correct answer. Out of 126 respondents, 23.8% witnessed cases of osteonecrosis of jaws in their workplace. About 62% (n=78) of the practitioners could correctly indicate the most appropriate definition of MRONJ. When the practitioners were questioned about the importance of dental consultation to remove all the oral foci of infection before the commencement of bone modifying agents drug therapy, only 49.2% (n=62) of them considered it a mandatory requirement. Approximately 33% (n=42) of them chose the option not required, 14.3% (n=18) were not sure and 3.2% did not respond. When the awareness regarding MRONJ guidelines and drug holiday concept was evaluated, only 8.7% (n=11) replied they were aware of the MRONJ management guidelines, 73% (n=92) were unaware, and the remaining (15.1%) were unsure of it.

Regarding the "drug holiday concept", 47.6% (n=60) were familiar with it. Overall, most participants (96%) felt the need for continuous medical education to keep them updated regarding these upcoming issues. Table 3 presents the difference in the responses based on their specialty.

**Table 3.** The difference in responses based on their specialty.

	Orthopedics	General/Internal Medicine	Oncologists	Total	Test statistic	P-value
**Do you think dental consultation is necessary for patients before starting them on bisphosphonates or any other antiresorptive antiangiogenic agents?**						
Yes/N (%)	29 (23.8%)	21 (17.2%)	12 (9.8%)	62 (50.8%)	12.65 ^a^	0.01 *
No/N (%)	31 (25.40%)	10 (8.2%)	1 (0.80%)	42 (34.4%)		
Not sure/N (%)	11 (9%)	7 (5.7%)	0 (0%)	18 (14.8%)		
Total/N (%)	71 (58.2%)	38 (31.10%)	13 (10.70%)	122 (100.0%)		
**Are you aware of MRONJ Guidelines?**						
Yes/N (%)	7 (5.7%)	2 (1.6%)	2 (1.6%)	11 (9.0%)	7.63 ^a^	0.08
No/N (%)	57 (46.7%)	25 (20.5%)	10 (8.2%)	92 (75.40%)		
Not sure/N (%)	7 (5.7%)	11 (9.0%)	1 (0.8%)	19 (15.60%)		
Total/N (%)	71 (58.2%)	38 (31.1%)	13 (10.7%)	122 (100.0%)		
**Are you aware of the drug holiday concept?**						
Yes/N (%)	29 (23.6%)	24 (19.50%)	7 (5.7%)	60 (48.80%)	4.94 ^b^	0.08
No/N (%)	42 (34.10%)	14 (11.40%)	7 (5.7%)	63 (51.2%)		
Not sure/N (%)						
Total/N (%)	71 (57.7%)	38 (30.90)	14 (11.40%)	123 (100%)		
**Have you come across osteonecrosis of the jaw as a side effect in patients on antiresorptive/antiangiogenic agents?**						
Yes/N (%)	11 (8.9%)	9 (7.3%)	10 (8.10%)	30 (24.4%)	19.85 ^b^	<0.001 **
No/N (%)	60 (48.8%)	29 (23.6%)	4 (3.30%)	93 (75.6%)		
Not sure/N (%)						
Total/N (%)	71 (57.70%)	38 (30.90%)	14 (11.40%)	123 (100%)		
**Do you feel there is a need to create awareness about MRONJ through Continuing Medical Educational programs?**						
Yes/N (%)	69 (56.10%)	38 (30.90%)	14 (11.40%)	121 (98.4%)	1.07 ^a^	0.64
No/N (%)	2 (1.6%)	0	0	2 (1.6%)		
Not sure/N (%)						
Total/N (%)	71 (57.70%)	38 (30.90%)	14 (11.4%)	123 (100%)		

Table 3 showes that orthopedics majorly felt it was unnecessary to consult dentists before starting bisphosphonates. General medicine and oncologists felt it is necessary to consult a dentist. The difference observed in the responses was statistically significant. The majority of participants were unaware of the MRONJ guidelines. The difference observed in the responses was not statistically significant. The "drug holiday concept" was less known to orthopedics. Among the general medicine doctors and oncologists, most of them were aware of it. Nevertheless, the difference in response was not statistically significant.

Regarding having witnessed cases of osteonecrosis, orthopedics and general medicine doctors had not come across osteonecrosis of the jaw compared to oncologists, and the difference was statistically significant. Participants, irrespective of their specialty, felt there was a need to create awareness about MRONJ. There was no statistically significant difference in the responses among different groups.

## DISCUSSION

MRONJ patients present with painful exposed bone/fistula or even in the form of non-exposed asymptomatic cases, having poor prognosis and reduced quality of life. Bone modifying agents like Bisphosphonates and Denosumab are principally used in long-term cancer treatment and in osteoporosis cases to reduce skeletal-related events (SRE) like metastasis and fracture [7]. The first case of MRONJ was reported in 2003, which was linked to the usage of bisphosphonates and, eventually, many other drugs were also found as the causative factor-like chemotherapeutic agents, angiogenesis inhibitors and tyrosine kinase inhibitors, mechanistic target of rapamycin inhibitors, BRAF inhibitors, and immune checkpoint inhibitors [8]. Bisphosphonate molecules anchor themselves in hydroxyapatite binding sites on the surface of the bone and hamper the osteoclastic activity, leading to apoptosis of the cells. Monoclonal antibodies like Denosumab impede the osteoclast formation by binding to the RANK ligand on the surface of the osteoclast precursor [1].

MRONJ is more prevalent in patients receiving high cumulative doses and high potency medications. About 90% of necrosis cases are seen in cancer patients receiving a high dose of IV therapy [1]. The pathogenesis of MRONJ is not fully explained, but local infection, inflammation, trauma with diminished bone repair capacity and inhibited angiogenesis, altered immunity are the most commonly elucidated factors. The occurrence of this potentially serious complication can be significantly reduced if holistic preventive measures from dentists, medical professionals, nursing and allied health professions are taken [9]. This study was conducted to evaluate the knowledge level about MRONJ and the implementation of dental referrals to patients being advised with BMAs.

In this study, 26% of the respondents were experienced professionals with more than 6 years of experience. Senior doctors were more aware of MRONJ, which can be due to their increased exposure to MRONJ cases compared to junior doctors. Also, doctors in tertiary care centers are more likely to know about MRONJ than doctors working in primary health centers. The medical practitioners who usually prescribed these drugs presented good academic knowledge about MRONJ; 61.9% of the respondents correctly identified the accurate definition of MRONJ. In contrast, less than half (*i.e.*, 47.6%) reported having an adequate understanding of the "drug holiday concept" for MRONJ prevention. A systematic review on MRONJ treatment reported that a patient who followed the "drug holiday period" showed significant complete healing of extraction sites [10]. Previous studies also reported similar results regarding the knowledge level [11, 12]. The benefit-risk ratio of using bone modifying agents varies from patient to patient based on the disease extent, location, and activity. By having a clear understanding and delivering the right information to patients regarding MRONJ, medical practitioners can play an exceptional role in turning the risk-benefit ratio of these medications [4].

Implementation of factual knowledge into clinical practice was also evaluated in the survey. More than half of the medical practitioners (63.5%) were prescribing BP about 0–5 times a month, mostly for osteoporosis cases. Even though a maximum number of participants reported prescribing these medications, only 49.2% of practitioners considered it important to seek a dental consultation and remove all sources of the oral nidus of infection before the commencement of BMAs therapy. Mohaya *et al.* reported similar findings, where more than half of the physicians who participated in the study never advised pre-treatment dental screening [11]. Previous studies reported that tooth extraction in patients on BMAs is one of the highest risk factors, and tooth extraction and oral infections were noted in 61.8% and 48.3% of patients with MRONJ [4, 13, 14]. A study on Lebanese physicians showed an alarming lack of knowledge regarding various aspects of BRONJ and suggested many more international group studies for spreading awareness among practitioners [9].

Another concerning factor was the management guidelines; even though 23.8% of the practitioners in the study witnessed patients with osteonecrosis as side effects of BMAs, most of them (73%) responded having inadequate knowledge about its management guidelines. Similar research conducted among dentists showed similar findings [15, 16]. The latest review states that teriparatide and mesenchymal stem cells therapy are novel treatment options for the management of MRONJ [17].

 Excellent cooperation amongst members of the multi-professional team, involving nurses, physicians, oral oncologists, and oral and maxillofacial surgeons, is required for the prevention and appropriate management of MRONJ. An important barrier to MRONJ prevention is poor patient knowledge or understanding of preventive methods. Hence, a medical practitioner must start the risk assessment for MRONJ before the start of BMAs. Low-risk patients should be advised for regular dental visits and maintain good oral health. Clear information on risk factors and preventative measures must be delivered to patients timely. Further, practitioners must also follow the step-wise protocol for preventing and managing MRONJ [4]. Recent guidelines also recommend mandatory dental screening of all patients, essentially high-risk patients undergoing removal of impacted teeth and teeth with poor prognosis, including identifying and treating all sources of odontogenic infection.

Also, we would like to put forward a few recommendations for medical professionals to regularly implement while advising BMAs and antiangiogenic agents. This can advocate preventive strategies for MRONJ.

The following preventive strategies are recommended for medical professionals prescribing bone modifying agents:

1. Not all patients with MRONJ will have bone exposure; there can be subclinical disease also. Hence, before medication initiation, the physician must schedule regular dental consultations for patients on bone modifying agents and antiangiogenic agents during the course and post medication phase;
2. The routine panoramic radiograph may not give all the information in early-stage manifestations of MRONJ. If the initial clinical assessment is indicative of MRONJ, advanced imaging like Cone Beam Computed Tomography is an option to be considered;
3. Non-nitrogenous bisphosphonates do not cause MRONJ. Hence, alternate drug options can be considered for high-risk patients;
4. Patients on IV bisphosphonates can have high-risk MRONJ precipitation even after many years (10-12 years) of stopping the medication. Hence, physicians should instruct the patients regarding these side effects to prevent MRONJ;
5. If taken for more than 2 years, oral bisphosphonates for routine osteoporosis treatment also present a risk for MRONJ;
6. MRONJ can occur spontaneously even without a history of dental extraction. Hence, periodic dental evaluation to rule out subclinical cases must be scheduled;
7. Not all bone exposures in patients with malignancy on bisphosphonates are MRONJ. Hence, it is important to rule out other causes like metastatic bone malignancies, fungal necrosis etc;
8. Bisphosphonates with tyrosine kinase inhibitors can have a cumulative effect on MRONJ precipitation. Hence, physicians can opt for alternative newer drug options in high-risk patients to prevent MRONJ;
9. The various treatment options available for MRONJ are conservative, minimally invasive, and surgical. Antibiotics do not cure MRONJ.

The present study had a few limitations, as the questionnaire was distributed through e-mails, the probability of response bias should be considered owing to informal discussions among the participants. Also, the small sample size and low response rate reported in the present study can be attributed to the reserved population in the chosen areas; hence, extrapolating the results to all physicians may not be precise.

## CONCLUSION

We observed that the majority of medical practitioners had adequate academic knowledge regarding MRONJ. However, there was a major gap in the knowledge-based clinical application. Even though 23.8% of participants had witnessed osteonecrosis cases, 73% of practitioners were not aware of the management guidelines. The lack of initiative to make referrals to the dentist before initiating drug therapy can be a pivotal factor in patient morbidity. Almost 33% of the practitioners in our study considered dental consultation as not a mandatory requirement. Even though the incidence of MRONJ is low, the pain and compromise in function greatly contribute to lowering the quality of life of this patient. We suggest that every medical practitioner prescribing BMAs should advise and remind patients about a regular dental visit, keep a check on risk factors like diabetic status, and monitor for symptoms of MRONJ. A standard referral pattern and a coordinated network should be established between dentists and medical professionals to prioritize patients on MRONJ inducing drugs. Further multicentric studies at the national level involving various specialties that prescribe bone modifying agents may be performed to develop a standardized protocol to address MRONJ.

## ACKNOWLEDGMENTS

### Conflict of Interest

The authors declare no conflicts of interest.

### Ethical approval

This study was approved by the Institutional Ethics Committee (IEC no 98/2018).

### Consent to participate

Written informed consent was obtained from participants.

### Personal thanks

The authors are grateful to all the medical practitioners for actively participating in the study.

### Authorship

SA, VP, and VR contributed to conceptualizing. SA, VP, VR, KN contributed to methodology, VP, SA contributed to writing the original draft. VR, AK contributed to editing the manuscript. SA, VP, contributed to data collection. SA, VP, VR contributed to data curation. KN contributed to data analysis.
